# Prehabilitation in degenerative cervical myelopathy: An underexplored opportunity to optimize outcomes

**DOI:** 10.1016/j.bas.2026.106105

**Published:** 2026-05-20

**Authors:** Maryse Fortin, Rohil V. Chauhan, Timothy F. Boerger, Chanelle Montpetit, Adam Kobaisi, Justin M. Lantz, Vishal Kumar, Joshua Plener, Andreas K. Demetriades, Lianne Wood, OZM Dastagir, Caroline Treanor, Georgios Toumbas, Carlo Ammendolia, Nicky Wilson, David B. Anderson, RECODE-DCM Perioperative Rehabilitation Incubator

**Affiliations:** a Department of Health, Kinesiology & Applied Physiology, Concordia University, Montreal, Quebec, Canada; b School of Health, Concordia University, Montreal, Quebec, Canada; c Department of Orthopaedic Physiotherapy, Auckland Spine Surgery Centre, Auckland, New Zealand; d School of Clinical Sciences, Faculty of Health and Environmental Sciences, Auckland University of Technology, Auckland, New Zealand; e Joint Department of Biomedical Engineering, Marquette University and the Medical College of Wisconsin, Milwaukee, WI, USA; f Division of Biokinesiology and Physical Therapy, Keck School of Medicine, University of Southern California, Los Angeles, USA; g Department of Orthopaedics, Postgraduate Institute of Medical Education & Research, Chandigarh, India; h Department of Anesthesiology and Pain Management, Mount Sinai Hospital, Toronto, Canada; i Edinburgh Spinal Surgery Outcome Studies Group, Department of Neurosurgery, Royal Infirmary Edinburgh, UK; j Department of Neurosurgery, Leiden University Medical Centre, Leiden, the Netherlands; k Department of Public Health and Sports Science, University of Exeter, Exeter, UK; l Department of Orthopaedic Surgery, Kuwait Bangladesh Friendship Government Hospital, Dhaka, Bangladesh; m National Neurosurgical Centre, Beaumont Hospital, Dublin, Ireland; n School of Physiotherapy, Royal College of Surgeons, Ireland; o Division of Neurosurgery, Department of Clinical Neurosciences, University of Cambridge, Cambridge, UK; p Department of Surgery, University of Toronto, Toronto, Canada; q Department of Physiotherapy, King's College Hospital NHS Foundation Trust, London, UK; r Sydney School of Health Science, Faculty of Medicine and Health, The University of Sydney, Sydney, Australia; s Sydney Spine Institute, Burwood, Sydney, Australia

Letter to Editor

While degenerative cervical myelopathy (DCM) is the most prevalent cause of non-traumatic spinal cord dysfunction in adults worldwide, it remains under-recognized in clinical practice. Globally, the average time from symptom onset to diagnosis is 15 months (Malone et al., 2025). DCM is marked by progressive spinal cord compression, neuronal apoptosis, and chronic inflammation, often leading to neurological deficits including gait and balance disturbances, hand dysfunction, and muscle weakness (Malone et al., 2025). In moderate to severe cases, surgical decompression is advised to stop further neurological deterioration (Fehlings et al., 2017). However, surgery alone does not address the associated physical deconditioning and musculoskeletal sequelae, such as reduced cervical mobility, impaired walking capacity, muscle weakness and atrophy, dexterity loss, and pain that accompany prolonged DCM. Furthermore, symptoms often persist following surgery, with 25-50% of patients experiencing minimal improvement; failing to achieve the minimally clinically important difference for functional or quality-of-life outcomes (Jaja et al., 2023). This raises an important question that remains largely unexplored: *could prehabilitation improve outcomes for individuals with DCM?*

Prehabilitation is defined as structured, multimodal interventions delivered preoperatively to optimize physical function, psychological wellbeing, nutritional health and medical comorbidities, with the aim of enhancing functional capacity and perioperative resilience (Gränicher et al., 2025; van Koningsveld-Couperus et al., 2025). Prehabilitation programs in patients undergoing orthopedic (total hip or knee replacement) and lumbar spinal surgery have demonstrated benefit in improving: functional recovery, complication rates, length of stay and quality of life (McIsaac et al., 2025; Punnosse et al., 2023). Given that individuals with DCM often present with muscle weakness, atrophy and dysfunction, impaired balance and reduced aerobic capacity (Chauhan et al., 2025), addressing these impairments - along with other modifiable predictors of postoperative outcomes (preoperative neck pain and smoking) (Jaja et al., 2023) - through prehabilitation may optimize function and improve post-operative outcomes.

While prehabilitation may offer theoretical benefits in DCM, some challenges must be considered. First, how DCM-related impairments influence free-living physical activity is not well established, representing an important gap for designing targeted prehabilitation. Second, surgical delays may lead to further neurological deterioration, which in turn can increase the complexity of prehabilitation. Lastly, exercise prescription must avoid exacerbating spinal cord compression (dynamic cervical loading), but limb strengthening and modified cervical exercise (isometric exercises) can likely be implemented safely. Even with these considerations, evidence demonstrating that prehabilitation improves postoperative recovery and functional outcomes in DCM is currently lacking.

Nevertheless, targeted resistance and aerobic training could counteract disuse atrophy, improve physical function, strength and neuromuscular efficiency, reduce the risks of falls and ultimately shorten hospital stays (Gränicher et al., 2025). Cervical muscle dysfunction (fatty infiltration and asymmetry) is also associated with poorer outcomes (Naghdi et al., 2023), including reduced functional recovery (mJOA, Nurick scores), persistent pain, and lower health-related quality of life, providing a rationale for individualized neuromuscular training. Furthermore, addressing modifiable factors beyond physical benefits, such as nutrition, and psychological factors may enhance surgical readiness and improve adherence to postoperative rehabilitation (Punnosse et al., 2023).

Therefore, a multimodal and personalized approach is likely necessary, combining targeted exercise, nutrition, education, psychological support and comorbidity management (van Koningsveld-Couperus et al., 2025; Punnosse et al., 2023). Early introduction of exercise through education and prehabilitation may boost confidence in muscle use and balance, promote exercise adherence and social participation, and ultimately enhance quality of life. Lifestyle modifications are an equally important component, particularly strategies for smoking cessation and alcohol use (van Koningsveld-Couperus et al., 2025; Chauhan et al., 2025). Such programs could be delivered in outpatient clinics/settings or remotely via tele-health ensuring accessibility for patients with mobility restrictions or those living in remote areas (van Koningsveld-Couperus et al., 2025).

However, as highlighted above, empirical evidence supporting the use of prehabilitation in DCM is virtually nonexistent (Smith et al., 2025). Best quality evidence primarily focuses on post-operative rehabilitation or surgical techniques, leaving a critical evidence gap for perioperative rehabilitation (Smith et al., 2025). This gap has been identified as a high research priority by the AO Spine RECODE DCM Perioperative Rehabilitation Incubator, an international multidisciplinary group advancing DCM research (Chauhan et al., 2025). Feasibility studies and pilot trials are urgently needed to determine acceptability, safety, adherence and preliminary efficacy. Key research questions include the optimal delivery, dose and duration of prehabilitation, the relative contribution of exercise, psychosocial support, education, nutrition and lifestyle modifications, as well as the influence of neurological severity on recovery trajectories.

Members of the RECODE-DCM Perioperative Rehabilitation Incubator are currently contributing to the development of Enhanced Recovery After Surgery (ERAS) guidelines for pre- and postoperative management of DCM and working on developing pilot trials to evaluate the feasibility and safety of prehabilitation in DCM. Alongside these efforts, a structured knowledge implementation plan could involve embedding prehabilitation protocols within pre-surgical assessment pathways, supported by clinician training, patient education resources, and evaluation of implementation outcomes (e.g., feasibility, acceptability and fidelity). We encourage the spine and rehabilitation research community to prioritize investigations in this area to address this critical gap. Collaborative efforts among surgeons, anesthesiologists, pre-assessment clinic nurses, primary care physicians, rehabilitation specialists (e.g., physical therapists, chiropractors), nutritionists and psychologists will be essential.

As the prevalence of DCM rises with an aging population, prehabilitation represents a critical opportunity to address modifiable impairments not adequately targeted by current care pathways. Despite the established benefits of prehabilitation in other surgical populations, there remains an urgent need for rigorous, high-quality research to determine its feasibility and effectiveness in DCM surgery and to inform evidence-based integration into clinical practice.

## Conflict of interest

The authors declare the following financial interests/personal relationships which may be considered as potential competing interests: Maryse Fortin reports a relationship with Concordia University that includes: employment. If there are other authors, they declare that they have no known competing financial interests or personal relationships that could have appeared to influence the work reported in this paper.
